# Inflammatory disease and C-reactive protein in relation to therapeutic ionising radiation exposure in the US Radiologic Technologists

**DOI:** 10.1038/s41598-019-41129-w

**Published:** 2019-03-20

**Authors:** Mark P. Little, Michelle Fang, Jason J. Liu, Ann Marie Weideman, Martha S. Linet

**Affiliations:** Radiation Epidemiology Branch, Division of Cancer Epidemiology and Genetics, National Cancer Institute, Bethesda, MD 20892-9778 USA

## Abstract

Chronic inflammation underlies many autoimmune diseases, including hypothyroidism, hyperthyroidism, and rheumatoid arthritis, also type-2 diabetes and osteoarthritis. Associations have been suggested of high-dose ionising radiation exposure with type-2 diabetes and elevated levels of C-reactive protein, a marker of chronic inflammation. In this analysis we used a proportional hazards model to assess effects of radiotherapy on risks of subsequent inflammatory disease morbidity in 110,368 US radiologic technologists followed from a baseline survey (1983–1989/1994–1998) through 2008. We used a linear model to assess log-transformed C-reactive protein concentration following radiotherapy in 1326 technologists. Relative risk of diabetes increased following radiotherapy (*p* < 0.001), and there was a borderline significant increasing trend per treatment (*p* = 0.092). For osteoarthritis there was increased relative risk associated with prior radiotherapy on all questionnaires (*p* = 0.005), and a significant increasing trend per previous treatment (*p* = 0.024). No consistent increases were observed for other types of inflammatory disease (hypothyroidism, hyperthyroidism, rheumatoid arthritis) associated with radiotherapy. There was a borderline significant (*p* = 0.059) increasing trend with dose for C-reactive protein with numbers of prior radiotherapy treatments. Our results suggest that radiotherapy is associated with subsequent increased risk of certain inflammatory conditions, which is reinforced by our finding of elevated levels of C-reactive protein.

## Introduction

Chronic inflammation underlies many autoimmune diseases, including hypothyroidism, hyperthyroidism, rheumatoid arthritis, type-1 diabetes and Crohn’s disease^1,2^. Type-2 diabetes and osteoarthritis are not auto-immune diseases, but also have a substantially inflammatory etiology^2–4^. Associations have been suggested of moderate/high dose ionising radiation exposure with certain inflammatory diseases, in particular with hypothyroidism in Chernobyl ^131^I-exposed populations^5,6^, with type-2 diabetes after high-dose radiotherapy (RT) of the abdomen in adults treated for peptic ulcer^7^, and with type-2 diabetes in persons treated for childhood cancer^8–10^. However, associations of high-dose radiation exposure with other inflammatory diseases have not been demonstrated.

One of the best-known markers of systemic inflammation is C-reactive protein (CRP), which is produced by the liver in response to acute inflammation^11^. Since CRP is rapidly metabolized, persistently elevated levels of CRP are a marker of continuing chronic inflammation. Dose-related excess levels of CRP have been observed in the Japanese atomic-bomb survivors Life Span Study (LSS) cohort^12^ and in persons receiving RT^13–16^.

In this paper we investigated associations of several types of inflammatory disease morbidity (specifically hypothyroidism, hyperthyroidism, type-2 diabetes, rheumatoid arthritis, osteoarthritis) and CRP with previous RT exposure. We evaluated exposure response using the proxy measure of numbers of treatments or number/type of irradiated body regions determined from the questionnaires that the technologists completed.

## Results

Table 1 shows that there were generally highly significant (*p* < 0.001) risks for all five morbidity endpoints associated with most of the underlying lifestyle and medical risk factors. Smoking was associated with excess risk of all outcomes except hypothyroidism and osteoarthritis. Race/ethnicity was linked to all outcomes except hyperthyroidism and rheumatoid arthritis. In consequence, all of these risk factors (sex, body mass index, smoking status, racial/ethnic group, smoking), year of birth, and duration of work were used as adjustment factors for the analysis presented in Table 2. Additional analysis given in Supplementary Information Part B Table B2 demonstrates the additional breakdown by RT and the significant variation that was thereby introduced. Supplementary Information Part B Table B2 demonstrates that there were significant departures from the random distribution of [case vs non-case] × [RT vs non-RT] in relation to almost all the putative risk factors (sex, racial/ethnic group, birth year, attained age, smoking status, BMI) that would be expected given the marginal totals.

**Table 1:** Distribution of numbers of informative inflammatory disease incident cases/numbers and relative risks according to demographic and lifestyle factors among 110,368 US Radiologic Technologists.

	Hypothyroidism			Hyperthyroidism			Type 2 diabetes		
	Hypothyroidism/no hypothyroidism	RR (+95% CI)^a^	*p*-value^a^	Hyperthyroidism/no hyperthyroidism	RR (+95% CI)^a^	*p*-value^a^	Diabetes/no diabetes	RR (+95% CI)^a^	*p*-value^a^
	Smoking status at baseline			Smoking status at baseline			Smoking status at baseline		
Non-smoker	2373/33,818	1 (=reference)	0.067	488/36,306	1 (=reference)	<0.001	1131/24,099	1 (=reference)	<0.001
Former smoker	1287/19,583	0.92 (0.86, 0.98)		291/21,012	1.04 (0.90, 1.20)		761/13,366	1.02 (0.93, 1.12)	
Current smoker	1023/15,454	0.94 (0.87, 1.01)		319/16,388	1.45 (1.26, 1.67)		638/9790	1.24 (1.12, 1.37)	
Unknown smoking status	42/585	1.05 (0.77, 1.42)		7/625	0.89 (0.42, 1.87)		27/323	1.34 (0.91, 1.96)	
	Body mass index (BMI) (kg/m^2^) at baseline			Body mass index (BMI) (kg/m^2^) at baseline			Body mass index (BMI) (kg/m^2^) at baseline		
18.5–24.9	3170/44,262	1 (=reference)	<0.001	755/47,249	1 (=reference)	<0.001	679/31,800	1 (=reference)	<0.001
missing	135/1692	1.14 (0.96, 1.35)		21/1835	0.74 (0.48, 1.14)		100/1050	3.87 (3.14, 4.77)	
<18.5	137/2186	0.91 (0.77, 1.08)		55/2292	1.52 (1.16, 2.00)		17/1516	0.59 (0.37, 0.96)	
25.0–29.9	919/15,883	0.81 (0.75, 0.87)		190/16,994	0.72 (0.61, 0.84)		1037/10,081	4.08 (3.70, 4.49)	
≥30.0	364/5417	0.96 (0.86, 1.08)		84/5961	0.93 (0.74, 1.17)		724/3131	8.73 (7.86, 9.70)	
	Racial/ethnic group			Racial/ethnic group			Racial/ethnic group		
White	4612/65,944	1 (=reference)	<0.001	1053/70,748	1 (=reference)	0.844	2350/45,792	1 (=reference)	<0.001
Black	42/1873	0.35 (0.26, 0.47)		29/1893	1.12 (0.77, 1.62)		121/943	2.13 (1.77, 2.55)	
Asian + Pacific islander	25/778	0.49 (0.33, 0.73)		12/794	1.08 (0.61, 1.91)		41/411	1.83 (1.34, 2.49)	
Other/unknown	46/845	0.78 (0.58, 1.04)		11/896	0.83 (0.46, 1.51)		45/432	2.03 (1.51, 2.73)	
	Sex			Sex			Sex		
Female	4317/52,143	1 (=reference)	<0.001	1023/56,647	1 (=reference)	<0.001	1681/38,934	1 (=reference)	<0.001
Male	408/17,297	0.29 (0.26, 0.32)		82/17,684	0.26 (0.21, 0.33)		876/8644	2.04 (1.88, 2.22)	
	Rheumatoid arthritis			Osteoarthritis					
	Arthritis/no arthritis	RR (+95% CI)^a^	*p*-value^a^	Osteoarthritis/no osteoarthritis	RR (+95% CI)^a^	*p*-value^a^			
	Smoking status at baseline			Smoking status at baseline					
Non-smoker	315/24,938	1 (=reference)	<0.001	1984/19,187	1 (=reference)	0.352			
Former smoker	225/13,998	1.11 (0.93, 1.31)		1212/9848	1.03 (0.96, 1.11)				
Current smoker	211/10,190	1.52 (1.27, 1.81)		866/7637	0.98 (0.91, 1.07)				
Unknown smoking status	8/342	1.48 (0.73, 2.98)		22/232	0.76 (0.50, 1.16)				
	Body mass index (BMI) (kg/m^2^) at baseline			Body mass index (BMI) (kg/m^2^) at baseline					
18.5–24.9	409/31,642	1 (=reference)	0.001	2536/24,671	1 (=reference)	<0.001			
missing	23/1134	1.42 (0.94, 2.17)		94/802	1.04 (0.85, 1.28)				
<18.5	21/1489	1.22 (0.79, 1.89)		95/1241	0.84 (0.69, 1.04)				
25.0–29.9	219/11,073	1.34 (1.14, 1.58)		892/7745	0.99 (0.92, 1.07)				
≥30.0	87/4130	1.43 (1.13, 1.80)		467/2445	1.60 (1.45, 1.77)				
	Racial/ethnic group			Racial/ethnic group					
White	727/47,440	1 (=reference)	0.265	3958/35,245	1 (=reference)	<0.001			
Black	21/1066	1.18 (0.76, 1.82)		87/825	0.84 (0.68, 1.04)				
Asian + Pacific islander	3/473	0.41 (0.13, 1.26)		18/407	0.39 (0.24, 0.61)				
Other/unknown	8/489	1.09 (0.54, 2.18)		21/427	0.44 (0.29, 0.68)				
	Sex			Sex					
Female	650/39,716	1 (=reference)	<0.001	3673/29,072	1 (=reference)	<0.001			
Male	109/9752	0.61 (0.50, 0.75)		411/7832	0.38 (0.34, 0.42)				

^a^The relative risk and *p*-values are obtained via fitting a Cox model with age as timescale, adjusted for year of birth (<1900, 1900–1909, 1910–1919, 1920–1929, 1930–1939, 1940–1949, 1950–1959, 1960+).

**Table 2:** Incidence of inflammatory disease following administration of radiotherapy in 110,368 U.S. radiologic technologists^a^.

Radiotherapy body regions received	Hypothyroidism		Hyperthyroidism		Type-2 Diabetes		Rheumatoid arthritis		Osteoarthritis	
	4725/74,165	*p*-value^b^	1105/75,436	*p*-value^b^	2557/50,135	*p*-value^b^	759/50,227	*p*-value^b^	4084/40,988	*p*-value^b^
	RR (95% CI)		RR (95% CI)		RR (95% CI)		RR (95% CI)		RR (95% CI)	
All radiotherapy (Q1)	1.03 (0.93, 1.13)	0.601	0.98 (0.79, 1.21)	0.852	1.29 (1.17, 1.43)	<0.001	0.93 (0.77, 1.12)	0.470	1.08 (0.99, 1.17)	0.092
All radiotherapy (Q1 + Q2)	1.12 (1.00, 1.26)	0.048	1.06 (0.84, 1.35)	0.623	1.07 (0.92, 1.24)	0.402	0.84 (0.63, 1.13)	0.232	1.19 (1.06, 1.35)	0.005
One vs None	1.05 (0.90, 1.22)	0.945	0.97 (0.70, 1.34)	0.983	1.07 (0.88, 1.32)	0.015	0.83 (0.55, 1.24)	0.387	1.26 (1.08, 1.48)	0.071
Two vs None	1.07 (0.72, 1.59)		0.84 (0.35, 2.03)		1.18 (0.74, 1.87)^c^		0.52 (0.13, 2.07)		1.11 (0.72, 1.71)	
Three vs None	0.91 (0.34, 2.44)		1.05 (0.15, 7.48)				3.06 (0.76, 12.37)		1.47 (0.61, 3.57)	
Four vs None	0.70 (0.17, 2.82)		1.58 (0.22, 11.29)		3.63 (1.48, 8.88)		2.04 (0.28, 14.65)		0.80 (0.20, 3.23)	
Continuous trend per number of treatments	1.02 (0.91, 1.13)	0.754^d^	0.98 (0.78, 1.23)	0.863^d^	1.12 (0.99, 1.28)	0.092^d^	0.96 (0.73, 1.26)	0.746^d^	1.14 (1.02, 1.27)	0.024^d^

^a^The relative risk and *p*-values are obtained via fitting a Cox model with age as timescale and adjusted, for duration of work (year last worked – year first worked), and via stratification by sex, year of birth (<1900, 1900–1909, 1910–1919, 1920–1929, 1930–1939, 1940–1949, 1950–1959, 1960+), body mass index (<18.5 or missing, 18.5–24.9, 25.0–29.9, 30.0+ kg m^−2^), smoking status (never, former, current smoker) and racial group (white, black, Asian, other/unknown). Unless otherwise indicated, all analysis uses only the responses to the first questionnaire (Q1) and not the second questionnaire (Q2).

^b^*p*-value of heterogeneity of relative risk, unless otherwise indicated;

^c^model with collapsed numbers of RT procedures: 0, 1, 2 + 3, 4;

^d^*p*-value of trend of relative risk with numbers of body regions receiving therapy.

Table 2 demonstrates indications of increased RR of type-2 diabetes with RT recorded at Q1 = 1.29 (95% CI 1.17, 1.43, *p* < 0.001). There was also a clear increasing dose response with increasing numbers of RT treatments, with RR increasing to 3.63 (95% CI 1.48, 8.88) for four treatments, and a borderline significant increasing trend per treatment, with RR/treatment = 1.12 (95% CI 0.99, 1.28, *p* = 0.092) (Table 2). Supplementary Information Part B Table B3 demonstrates elevated risk of diabetes with RT cancer treatment, with RR = 1.59 (95% CI 1.01, 2.52, *p* = 0.063). For Q1 data there was highly significant (*p* < 0.001) interaction of RT and BMI, with diabetes risk markedly elevated for those with BMI < 18.5 kg m^−2^; there was no such significant interaction for the combined (Q1/Q2) RT data, or for number of RT treatments (*p* > 0.5) (results not shown). Supplementary Information Part B Table B3 demonstrates elevated risk of rheumatoid arthritis associated with pelvic RT, with RR = 2.20 (95% CI 1.04, 4.67, *p* = 0.068). For osteoarthritis, there was increased RR associated with RT at Q1/Q2 = 1.19 (95% CI 1.06, 1.35, *p* = 0.005). There was a significant increasing trend per treatment, with RR/treatment = 1.14 (95% CI 1.02, 1.27, *p* = 0.024) (Table 2). Supplementary Information Part B Table B3 demonstrates elevated risk of osteoarthritis associated with head RT, with RR = 1.28 (95% CI 1.05, 1.55, *p* = 0.017) and with RT treatment for non-malignant disease, with RR = 1.25 (95% CI 1.06, 1.48, *p* = 0.013). No consistent increases were observed for other types of inflammatory disease associated with RT. These findings were generally similar in the minimally adjusted analysis (Supplementary Information Part B Table B1).

Table 3 shows that the distribution of CRP varies statistically significantly (*p* < 0.001) by sex, race and BMI, but not markedly for any of the other variables evaluated (birth year, age at blood draw, smoking). Sex and BMI were also among the variables chosen by step-AIC for inclusion in the optimal background model for CRP, along with the other variables listed in Supplementary Information Part A Table A2. Likewise, Supplementary Information Part B Table B4 shows the distribution of CRP in relation to lifestyle and medical variables, as well as RT. Supplementary Information Part B Table B4 demonstrates that there were significant departures (*p* < 0.05) from the random distribution of CRP level [<1 mg/l vs 1–<3 mg/l vs ≥3 mg/l] × [RT vs non-RT] in relation to sex, race, and BMI that would be expected given the marginal totals. However, there were no such significant departures for the other three variables (birth year, attained age, smoking status).

**Table 3:** CRP according to demographic and lifestyle factors, among 1326 US Radiologic Technologists with a usable blood draw sample^a^.

	CRP mean (mg/ml) (1^st^ quartile/median/3^rd^ quartile)	Relative excess of CRP compared with reference level (+95% CI)^b^	*p*-value^b^
**Sex**			
Female	5.66 (1.14/2.67/6.61)	1 (=reference)	<0.001
Male	4.07 (0.96/2.03/4.42)	0.76 (0.67, 0.87)	
**Racial group**			
White	4.43 (0.97/2.12/5.17)	1 (=reference)	<0.001
Black	6.12 (1.31/3.07/7.66)	1.35 (1.19, 1.55)	
Asian + Pacific islander	2.24 (0.84/1.62/3.64)	0.47 (0.12, 1.83)	
Other/unknown	2.64 (1.32/1.65/3.45)	0.54 (0.21, 1.41)	
**Birth year**			
<1920	4.18 (1.34/2.72/5.12)	1 (=reference)	0.142
1920–1929	4.70 (1.01/2.06/5.42)	0.83 (0.49, 1.42)	
1930–1939	5.41 (1.13/2.58/6.36)	0.98 (0.59, 1.63)	
1940–1949	5.55 (1.17/2.65/6.33)	1.00 (0.60, 1.66)	
≥1950	4.64 (0.95/2.23/5.70)	0.83 (0.50, 1.38)	
**Age at blood draw**			
<50	5.67 (0.55/1.57/6.10)	1 (=reference)	0.497
50–59	4.62 (1.03/2.29/5.81)	1.17 (0.64, 2.13)	
60–69	5.54 (1.11/2.56/6.28)	1.33 (0.72, 2.43)	
70–79	5.21 (1.13/2.29/6.39)	1.30 (0.71, 2.38)	
≥80	5.14 (1.13/2.25/5.35)	1.22 (0.64, 2.31)	
**Smoking**			
Not current smoker	5.03 (1.04/2.28/6.10)	1 (=reference)	0.174
Missing/unknown	5.42 (1.27/2.38/6.54)	1.14 (0.92, 1.40)	
Current smoker	4.67 (1.68/3.78/5.63)	1.35 (0.91, 2.01)	
**Body mass index (BMI) (kg/m** ^**2**^ **) at Q1**			
18.5–24.9	4.32 (0.91/2.05/5.31)	1 (=reference)	<0.001
unknown	5.63 (1.05/2.16/6.30)	1.14 (0.95, 1.35)	
<18.5	2.90 (0.82/1.64/2.78)	0.74 (0.44, 1.24)	
25.0–29.9	5.50 (1.41/2.93/6.51)	1.36 (1.16, 1.60)	
≥30.0	6.90 (1.70/4.15/10.25)	1.82 (1.44, 2.30)	

^a^Information generally determined from the special questionnaire administered to the technologists at time of blood draw [RQ1], unless otherwise indicated – see Supplementary Information Part A Table A1.

^b^Relative excess CRP values over reference level, and *p*-value of significance of fit, established via fitting a linear model to the log-transformed CRP data.

**Figure 1:**
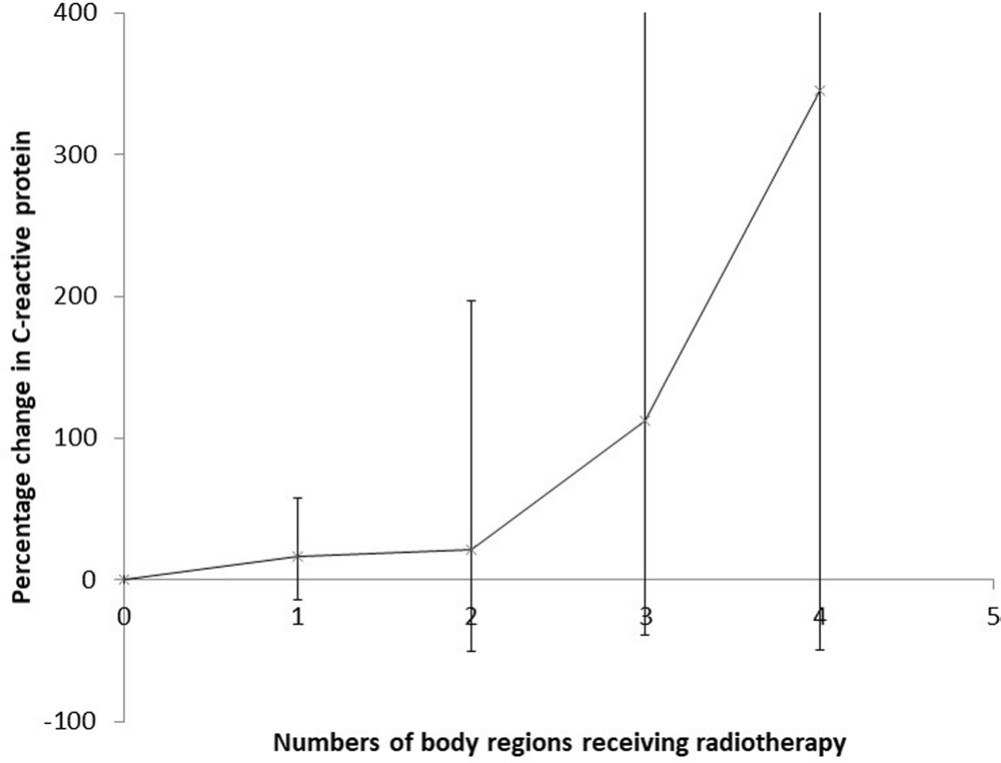
Percentage change (+95% CI) in C-reactive protein level with number of radiotherapy treatments. Analysis is as in Table 4.

Table 4 and Fig. 1 suggest that there was a borderline significant (*p* = 0.059) increasing trend with dose for CRP with numbers of RT treatments or body areas exposed to RT procedures reported, with CRP increasing by 21.4% (95% CI -0.7, 48.5) per RT procedure.

**Table 4:** Percent change in C-reactive protein (CRP) in relation to administration of radiotherapy (as a patient), among 1326 US Radiologic Technologists with a usable blood draw sample.

Number of radiotherapy treatments	Number of persons undergoing radiotherapy	Percent change in C-reactive protein from reference category (95% CI)	*p*-value
**Total number of radiotherapy treatments**			
0	1260	0 (ref)	0.491^a^
1	56	16.5 (−13.7, 57.4)	
2	6	21.4 (−50.3, 196.5)	
3	3	112.6 (−38.9, 639.8)	
4	1	344.8 (−49.9, 3846.2)	
**Continuous trend per number of radiotherapy treatments**			
	66	21.4 (−0.7, 48.5)	0.059^b^

Analysis is adjusted for step-AIC optimal background variables given in Supplementary Information Part A Table A2, using combined radiotherapy treatment data from 1^st^ and 2^nd^ questionnaires.

^a^*p*-value of heterogeneity of relative risk;

^b^*p*-value of trend of relative risk with numbers of treatments.

## Discussion

This large and predominantly female cohort is the first prospective cohort study to identify an association of RT with subsequent report of osteoarthritis and only the second known study to link RT in adulthood with type-2 diabetes. A potential mechanism for these associations is suggested by the marginally significant (*p* = 0.059) finding of increased levels of CRP, a marker of systemic chronic inflammation, associated with RT. There were no indications of effects of RT on other types of inflammatory disease, specifically hypothyroidism, hyperthyroidism, or rheumatoid arthritis. A novel feature of this study is examination of various type of inflammatory disease in relation to numbers of RT treatments.

There have been only four previous studies examining the relationship between type-2 diabetes and high dose RT. Kleinerman *et al*.^7^ found a RR of 3.79 (95% CI 1.2, 12.0) associated with >16 Gy to the pancreas in a group treated for peptic ulcer in adulthood. In a Dutch cohort of childhood cancer survivors a dose of ≥36 Gy to the pancreas tail was associated with an RR of 2.43 (95% CI 1.22, 4.85)^10^. In a French-UK cohort of childhood cancer survivors^8^, there was an excess RR of 0.61 Gy^−1^ (95% CI 0.21, 1.68) associated with dose to the pancreas tail. Because doses were not estimated in our study, it is difficult to compare our risks with these studies. In another cohort of childhood cancer survivors, Meacham *et al*.^9^ documented an RR associated with abdominal irradiation of 3.4 (95% CI 2.3, 5.0), consistent with our estimated RR of 1.71 (95% CI 0.85, 3.47) associated with abdominal RT (Supplementary Information Part B Table B3). To the best of our knowledge, for none of the other inflammatory endpoints studied have there been assessments of the effects of prior RT.

The null findings for hypothyroidism and RT (Table 2) are consistent with the lack of dose-related prevalence in the LSS cohort^17^, although perhaps inconsistent with the dose-related excess observed in two Chernobyl-exposed cohorts^5,6^. It is likely that this apparent discrepancy is explained by the differences in type of radiation, with the current study implicitly examining external radiation from RT whereas the Chernobyl-exposed groups were exposed primarily to ^131^I^5,6^, but the discrepancy may also derive from the fact that in the Chernobyl-exposed groups the excess was largely restricted to sub-clinical disease, and possible differences in diagnostic criteria. The null findings for hyperthyroidism are consistent with the similarly null findings in the LSS cohort^17^ and in a Chernobyl-exposed group^6^.

There is evidence from prospective studies that elevated levels of the pro-inflammatory cytokines interleukin-6, CRP and tumor necrosis factor-α (TNFα) may be associated with type-2 diabetes^18,19^. There is also evidence of up-regulation of the anti-inflammatory interleukin-1 receptor antagonist, produced by the body in response to the pro-inflammatory interleukin-1β, before the onset of diabetes^20,21^.

The two best established risk factors for osteoarthritis are age and obesity^22^. Experimental studies have suggested mechanisms of cartilage degradation and reduced proteoglycan synthesis^23^. Excess occupational risks have also been associated with female cleaners and workers in clothing industries, male masons and other construction workers, and agricultural workers of both sexes^24^, possibly related to the element of heavy manual work in these professions. Many radiologic technologists wore leaded aprons, which would impose substantial load on the joints in the lower part of the body, although it is unlikely that use of such aprons would correlate with RT. Our analysis adjusted for numbers of years worked, which suggests that at least this crude cumulative measure of joint “wear” does not explain our findings. However, purely mechanical joint loading is only part of the picture. There is a considerable body of evidence that, in obese people, even non-weight bearing joints are affected by osteoarthritis, suggesting a role for lipid metabolism, and specifically certain adipokines, in causing osteoarthritis^3,25^. There is more general evidence that inflammatory cytokines such as interleukin-1, TNFα, and prostaglandin E_2_ play a role in rheumatoid arthritis and osteoarthritis^2^.

In view of the inflammatory etiology of osteoarthritis, it is unsurprising that type-2 diabetes, a largely inflammatory disease^4^, has been shown to be a risk factor (independent of BMI and age)^26,27^. It is also to be expected that osteoarthritis may be a risk factor for type-2 diabetes^28^, and more generally for metabolic syndrome^29^, of which type-2 diabetes is part. The relationship we found between RT and osteoarthritis is therefore not too surprising, given the inflammatory nature of the disease. There are indications from various case series that RT may be a risk factor for osteoarthritis^30–33^, but to the best of our knowledge this has not been established in any prospective cohort.

One of the best-known markers of inflammation is CRP, which is produced by the liver in response to acute inflammation, in particular the rise in concentration of the pro-inflammatory IL-6^11^. Dose-related excess levels of CRP have been observed in the LSS, 50–52 years after exposure^12^, and in persons receiving RT^13–16^, generally sampled a much shorter period (at most weeks) after RT. A weakness of all the studies of CRP associated with RT, as with our study, is that it is unclear whether it is the underlying disease for which RT is given, or the RT itself, that may be associated with elevated CRP. The finding that CRP was chronically elevated after RT is consistent with long-lasting dose-related change in CRP in the LSS cohort^12^, although the magnitude was much greater here (Table 4, Fig. 1) and in other groups given RT^13–16^, possibly a function of the likely much higher RT doses.

Strengths of this study include large size, prospective design, and availability of data on many covariates associated with all inflammatory endpoints, including obesity (via BMI), cigarette smoking and racial group. Adjustment for these variables had only minimal effect on risks of inflammatory disorders arising after RT; the minimally-adjusted and fully-adjusted RRs were very similar (Table 2, Supplementary Information Part B Table B1).

Weaknesses of the study include ascertainment of RT and inflammatory disorders solely by questionnaire and lack of validation by medical records. There was no information on location of osteoarthritis, in relation to the RT-treated areas. However, as discussed above, osteoarthritis is in part systemic, and so lack of locational information may be irrelevant. There is little information on timing of the RT procedures, so that these exposures could only be treated as non-time-varying in the analysis. However, the sensitivity and specificity of self-reported diagnosis of osteoarthritis in the general population are generally high, >75%^34–36^, while for type-2 diabetes the sensitivity and specificity are generally even higher, >80%^37–40^. A variety of studies, reviewed elsewhere^41^, suggest that self-reported rheumatoid arthritis is reported with variable sensitivity and specificity, between 10–90%. A Danish study suggests that self-reported hyperthyroidism and hypothyroidism are reported with high (98%) sensitivity, although only moderate (57–67%) specificity^42^. Recall of RT in the general population has been found to have a sensitivity of 60% or better, and specificity of 70% or better^43,44^. The population of radiologic technologists reported here was medically literate, so that self-diagnosis of osteoarthritis and type-2 diabetes and the location of RT fields should be even better than these figures would suggest. As with many occupational studies, cohort members had to survive to answer the first questionnaire. However, such selection will not necessarily bias our analysis, since everyone had to survive to answer a questionnaire, and risk was assessed conditional on that.

In summary, we found indications of increased risk of type-2 diabetes and osteoarthritis, and elevated levels of CRP associated with RT exposure. The findings for osteoarthritis are novel and in need of replication in other prospectively-evaluated cohorts. Future large prospective studies are needed with validation of RT using radiation oncology records that provide information about anatomic site and number of treatments and medical record validation of a broad range of inflammatory conditions with detailed information about onset and natural history.

## Data and Methods

### Overview

#### Study population and follow-up

The USRT study population and methods are described elsewhere^45–47^ and detailed information can be found online (www.radtechstudy.nci.nih.gov). Briefly, in the mid-1980s, the US National Cancer Institute, in collaboration with the University of Minnesota and the American Registry of Radiologic Technologists (ARRT), began a study of cancer incidence and mortality among 146,021 (106,953 women) US radiologic technologists who were certified for at least 2 years between 1926 and 1982^48^. Annual follow-up was conducted by obtaining records of yearly re-certification with the ARRT. For technologists who did not recertify, vital status through December 31, 2008 was obtained through periodic linkage with the Social Security Administration, and for those determined or presumed to be deceased, the cause of death was obtained through linkage with the National Death Index (NDI-*Plus*) records.

#### Data collection

The cohort was surveyed three times between 1983 and 2005. The first questionnaire (1983–1989)(Q1) was mailed to 132,298 known-living radiologic technologists, of whom 90,305 (68%) responded. The second questionnaire (1994–1998)(Q2) was mailed to 126,628 known-living technologists, of whom 90,972 (72%) responded. Both surveys asked for detailed work history information about employment as a radiologic technologist, lifestyle and other risk factors for cancer and other chronic diseases, and personal history of therapeutic medical radiation procedures. Technologists were also queried about personal history of cancer and selected other health outcomes associated with radiation exposure in other populations. The third questionnaire (2003–2005)(Q3) was mailed to 101,694 living cohort members who had completed at least one of Q1 or Q2; 73,838 technologists (73%) responded. Q3 elicited similar information on medical outcomes as well as detailed work history. In each of the three main questionnaires, participants were asked if they had ever been diagnosed with hypothyroidism or hyperthyroidism, and in Q2 and Q3 they were asked about type-2 diabetes, rheumatoid arthritis and osteoarthritis (Supplementary Information Part B).

### Eligible populations

#### Questionnaire assessment component

To be included in the current study, technologists had to have completed Q1 or Q2 and been followed through the earlier of either date of death or December 31, 2008 (the end of the study period). Of the 110,374 technologists meeting these criteria, the date of last vital status was unknown for five participants, and another individual had entry date equal to exit date; exclusion of these left an analysis dataset of 110,368 technologists. Demographic and lifestyle characteristics of the eligible population for this component are summarized in Table 1.

#### CRP component

Blood serum samples were taken from a subcohort of 1644 US radiologic technologists, selected from a random sample of the main USRT population that was enriched by inclusion of blacks and stratified by sex, age, and latitude, for a study of determinants of vitamin D^49,50^. Samples were obtained during 2008–2009, with a supplemental questionnaire (RQ1) concurrently administered. CRP was one of the determinants measured^49^. Of these 1644 technologists, 1327 (824 female) yielded detectable CRP. Methods for selecting the sample and processing biological material are described at greater length elsewhere^49,50^. One subject was removed from the dataset because the number of self-recorded body regions exposed to RT (11) substantially exceeded the maximum (of 4) in the remaining data, resulting in an analysis dataset of 1326 individuals. The demographic and clinical data associated with the blood draw participants originated from Q1 and Q2, the supplemental questionnaire administered concurrently with phlebotomy (RQ1), and a third full-length questionnaire that was mailed or administered by phone.

#### Outcomes selected for study

The five specific inflammatory disease outcomes selected were elicited at baseline and in the two follow-up questionnaires, as indicated above. Other inflammatory disease outcomes that technologists were asked about in follow-up, but not the baseline questionnaires, were excluded. For each morbidity endpoint, the subject had to have been free of the specified disease at study entry (as indicated by responses to questions about diagnosis of these conditions on Q1/Q2) and informative at the last questionnaire.

#### Exposure assessment

Questions were asked on Q1/Q2 about anatomic sites treated with RT, and additionally (a) on Q1 the first year treated and the number of treatments, and (b) on Q2 whether RT had been given by decade (<1980, 1980–1989, 1990+) and whether for cancer or other conditions.

#### Potential confounders

A literature review by AMW/MF suggested a number of variables potentially associated with CRP including age at blood draw, race, gender, and menopause-related variables, use of oral contraceptives, cigarette smoking, body mass index (BMI), exercise, various diseases potentially associated with an inflammatory mechanism (including inflammatory bowel disease, goiter, thyroiditis, hyperthyroidism, hypothyroidism, other thyroid disease, parathyroid disease (including hyperparathyroidism), angina, stroke, transient ischemic attacks, type-2 diabetes, Parkinson’s disease, scleroderma, etc). The complete list of variables used is given in Supplementary Information Part A Table A1.

Likewise, the literature review suggested that cigarette smoking is a risk factor for many of the inflammatory diseases studied^51–55^, similarly for obesity^55–57^. Racial and ethnic group is also a risk factor for type-2 diabetes^58^. It is likely that osteoarthritis may correlate with numbers of years worked, because of the requirement that technologists wear leaded aprons, with the consequent loading of torso and lower limbs. For these reasons all Cox regression analyses of the inflammatory endpoints were adjusted for number of years worked [= year last worked – year first worked], and by stratification for sex, year of birth, cigarette smoking at baseline, baseline BMI, and racial group, as shown in Table 2. Additional minimally-adjusted analysis is given in Supplementary Information Part B Table B1.

#### Statistical Methods

*Analysis of inflammatory disease and radiotherapy:* We evaluated history of RT at baseline (Q1/Q2) and then followed the subjects for subsequent incidence of the inflammatory diseases as reported in a questionnaire after baseline. We assessed the relationship between known and suspected risk factors for the incident inflammatory diseases (Table 1) and compared the frequency of RT in those reporting and those not reporting diagnosis of the incident inflammatory disease outcomes (Supplementary Information Part B Table B2). We used Cox proportional hazards models^59^ with age as the timescale to estimate relative risks (RR) in relation to numbers of RT procedures (Table 2) and by body part irradiated (Supplementary Information Part B Table B3). For all disease endpoints, follow-up started at the first questionnaire for which the endpoint was reported as absent, and continued until the earlier of the last questionnaire on which the morbidity endpoint was reported, or the reported date of onset of the endpoint in question. All models were fitted in R^60^ via maximization of the partial likelihood^59,61^. All *p*-values and confidence intervals were two-sided and likelihood based^61^. Further details on statistical methods are given in Supplementary Information Part B.

*Analysis of CRP and radiotherapy:* Measurement of CRP was undertaken in 2008–2009, 10–25 years after the baseline surveys. The primary aim of the study was to identify if there were significant changes in CRP with number of therapeutic radiation cycles by fitting a linear model to log-transformed CRP data (transformed to normalize the residuals) in relation to indicators of RT exposure. In order to determine which of the candidate variables (Supplementary Information Part A Table A1) affected CRP in this cohort, and to avoid over-fitting, the Akaike Information Criterion (AIC)^62,63^ was used to select the optimal variables (Supplementary Information Part A Table A2) from these. All statistical analyses were carried out using ordinary least squares, and tests were performed using analysis of variance (ANOVA)^64^ in R^60^. Further details on statistical methods are given in Supplementary Information Part B.

#### Ethical approvals

Informed consent was obtained from all participants. The research protocol for the USRT cohort study of cancer and other health risks has been approved annually by the National Cancer Institute Special Studies Institution Review Board (SSIRB; Protocol OH97-C-N053) and the University of Minnesota Human Research Protection Program Institution Review Board (Study number 8005M02489). All methods were performed in accordance with the relevant guidelines and regulations of the National Institutes of Health and the University of Minnesota.

## Supplementary information

Revised supplementary material file

## Data Availability

The data used is available from the principal author upon request.
