# Supplementary material for: Inflammatory disease and C-reactive protein in relation to therapeutic ionising radiation exposure in the US Radiologic Technologists
Source: Sci Rep. 2019 Mar 20;9:4891. doi: 10.1038/s41598-019-41129-w (PMC6426979; doi:10.1038/s41598-019-41129-w)
Supplement: Supplementary file 1 — Revised supplementary material file [file 41598_2019_41129_MOESM1_ESM.docx]

**Supplementary Information**

**Inflammatory disease and C-reactive protein in relation to therapeutic ionising radiation exposure in the US Radiologic Technologists**

Mark P Little, Michelle Fang, Jason J Liu, Ann Marie Weideman, Martha S Linet

**Supplementary Information Part A. Tables describing variables potentially and actually used to adjust C-reactive protein data**

**Supplementary Information Part A Table A1. Candidate variables used in step-AIC model fits to C-reactive protein data.** The questionnaire [Q1-first questionnaire, Q2-second questionnaire, Q3-third questionnaire, RQ1-special questionnaire administered at time of blood draw] from which the information was derived is also given.

| **Description** |
| --- |
| **Demographic** |
| Gender |
| Age at first blood draw |
| Racial group |
| **Menopause-related** |
| Have menstrual periods stopped completely? [RQ1] |
| Age at menopause [Q2] |
| **Oral contraceptives and other hormonal modifiers** |
| Ever taken diethyl stilboestrol (DES) [Q2] |
| Ever use hormone replacement therapy (HRT) [Q2] |
| Ever had menopause [Q2] |
| Ever take oral contraceptives [Q2] |
| Currently using hormone therapy? [RQ1] |
| Ever used oral contraceptives?,[RQ1] |
| First year hormone therapy used [RQ1] |
| Currently using HRT [Q2] |
| Ever used Progestins [Q2] |
| Mother ever use DES [Q2] |
| **Smoking** |
| Number of pack-years smoked [Q1] |
| Number of years smoked [Q1] |
| Number of cigarettes smoked per day [Q1] |
| Years smoked (nonsmokers identified) [Q2] |
| Pack-years smoked (nonsmokers identified) [Q2] |
| Number of cigarettes smoked per day (nonsmokers identified) [Q2] |
| Cigarettes per day usually smoked [Q3] |
| Past 30 days, amount cigarettes smoked per day [RQ1] |
| Do you smoke currently? [RQ1] |
| **Body mass index** |
| Body Mass Index (kg/m^2^) [Q1] |
| Body mass index (kg/m^2^) [Q2] |
| BMI (continuous) [RQ1] |
| **Exercise** |
| Exercise strenuously, hours per week [Q2] |
| Walking or hiking, hours per week [Q2] |
| Walking at home or work, hours per week [Q2] |
| # of stairs climbed daily, previous year [Q2] |
| **Inflammatory disease** |
| Ever diagnosed with inflammatory bowel, celiac disease, or bowel resection? [RQ1] |
| Ever have goiter (enlarged thyroid) [Q3] |
| Ever have thyroiditis (Hashimoto's Disease) [Q3] |
| Ever have hyperthyroidism (overactive thyroid) [Q3] |
| Ever have hypothyroidism (underactive thyroid) [Q3] |
| Ever have any other thyroid conditions (excluding adenoma, nodule, goiter, thyroiditis, hyperthyroidism, hypothyroidism) [Q3] |
| Ever have adenoma of the stomach [Q3] |
| Ever have pituitary tumor [Q3] |
| Ever have a benign tumor of the parathyroid gland [Q3] |
| Ever have hyperparathyroidism [Q3] |
| Ever have a colon polyp [Q3] |
| Ever have uterine fibroids (myoma) [Q3] |
| Ever have a benign tumor of the ovary [Q3] |
| Ever have fibrocystic or other benign breast disease [Q3] |
| Ever have high blood pressure [Q3] |
| Ever have stroke (cerebrovascular accident) [Q3] |
| Ever have TIA (transient ischemic attack) [Q3] |
| Ever have heart attack, myocardial infarction (MI) [Q3] |
| Ever have angina pectoris [Q3] |
| Ever have coronary bypass surgery [Q3] |
| Ever have macular degeneration [Q3] |
| Ever have cataracts [Q3] |
| Ever have glaucoma [Q3] |
| Ever have hepatitis [Q3] |
| Ever have cirrhosis of the liver [Q3] |
| Ever have an ulcer [Q3] |
| Ever have diabetes [Q3] |
| Ever have gallbladder problems [Q3] |
| Ever have multiple sclerosis [Q3] |
| Ever have Parkinson's disease [Q3] |
| Ever have attention-deficit disorder [Q3] |
| Ever have systemic lupus erythematosus [Q3] |
| Ever have osteoarthritis [Q3] |
| Ever have rheumatoid arthritis [Q3] |
| Ever have scleroderma [Q3] |
| Ever have stroke or TIA [Q3] |
| **Asthma** |
| Ever have diagnosis of Asthma [Q2] |
| **High cholesterol** |
| Ever have diagnosis of Cholesterol, Elevated (>= 240) [Q2] |
| **Non-steroidal anti-inflammatory drugs (NSAID) use** |
| Average days per month you took Aspirin (Anacin, Bufferin, etc.) [Q2] |
| Average days per month you took Other anti-inflammatory (Ibuprofen, Motrin, etc.) [Q2] |

**Supplementary Information Part A Table A2. Optimal explanatory variables from step-AIC model fits to C-reactive protein data (in order of selection to model).** The questionnaire [Q1-first questionnaire, Q2-second questionnaire, Q3-third questionnaire, RQ1-special questionnaire administered at time of blood draw] from which the information was derived is also given.

| **Description** |
| --- |
| Ever have high blood pressure [Q3] |
| BMI (continuous) [RQ1] |
| Gender |
| Ever have fibrocystic or other benign breast disease [Q3] |
| Ever have multiple sclerosis [Q3] |
| Ever have diabetes [Q3] |
| Past 30 days, amount cigarettes smoked per day [RQ1] |
| Age at menopause [Q2] |
| First year hormone therapy used [RQ1] |
| Ever used oral contraceptives? [RQ1] |
| Ever have any other thyroid conditions (excluding adenoma, nodule, goiter, thyroiditis, hyperthyroidism, hypothyroidism) [Q3] |
| Age at first blood draw |

**Supplementary Information Part B. Detailed statistical methods, wording of questions asked, and supplementary analyses**

*Analysis of inflammatory disease and radiotherapy*

Exposures of various parts of the body (head, pelvis, chest, extremity, shoulder, abdomen, other, total number of RT procedures) to RT were coded by variables$(RT_{ij})_{j=1}^{n}$, as determined by the questions asked on Q1 and Q2 (see below). We constructed variables for RT exposure which for each technologist took account of all information on such procedures before the time of entry of the subject into the analysis dataset. Specifically, for those persons entering the study before 1990 we only used questions relating to possible radiotherapy exposure from the first questionnaire, or pre-1980 parts of the second questionnaire. Morbidity risks in relation to RT in the cohort were assessed using a Cox proportional hazards model [^1^](#_ENREF_1), in which the relative risk (RR) for individual $i$ was given by:

$RR_{i}=\exp\left[\sum_{j=1}^{n} \beta_{j}RT_{ij}\right]$ (B1)

Risk in relation to region of body irradiated (head, pelvis, chest, extremity, shoulder, abdomen, other), and by numbers of RT procedures are reported in Table 2 and Supplementary Information Part B Table B3. For a more limited set of body regions for which information was elicited in the first questionnaire we report risks in relation only to these. For most analyses age was used as the timescale, with stratification by sex, year of birth (using categories of year of birth <1900, 1900-1909, 1910-1919, …, 1950-1959, ≥1960), cigarette smoking at baseline (never smoked/former smoker/current smoker), baseline body mass index (BMI) (kg m^-2^) (categorised as unknown + <18.5, 18.5-24.9, 25.0-29.9, ≥30.0), and racial group (white/black/Asian/other+unknown), and adjusting for number of years worked, reported in Table 2. Most of these variables are standard risk factors for many of these endpoints, but their effect was also assessed in Table 1. Years of work was adjusted for in part to control for a possible differential healthy worker effect, also the effect of cumulative “wear”, for example joint “wear” resulting from repetitive use of leaded aprons, with the consequent loading of torso and lower limbs, in relation to osteoarthritis. Sensitivity analyses were also conducted using a minimal set of adjustments, with stratification for sex and year of birth and adjusting for number of years worked, reported in Supplementary Information Part B Table B1. For all disease endpoints, follow-up started at the first questionnaire for which the endpoint was reported as not present, and continued until the earlier of the last questionnaire on which the morbidity endpoint was reported, or the reported date of onset of the endpoint in question.

All models were fitted via maximization of the partial likelihood [^1^](#_ENREF_1), using R [^2^](#_ENREF_2). All *p*-values and confidence intervals were two-sided and profile-likelihood based [^3^](#_ENREF_3) unless otherwise indicated. For the descriptive analysis in Supplementary Information Part B Table B2, heterogeneity *p*-values (recording departures from randomness conditional on the marginal totals) were computed using Fisher’s exact test [^4^](#_ENREF_4), or, when this was not possible, using an approximation based on 10^7^ simulations.

*Analysis of CRP and radiotherapy*

The primary aim of the study was to identify if there were significant changes in C-reactive protein (CRP) with location and increased number of therapeutic radiation cycles. To this end, we modelled CRP in relation to indicators of RT exposure. Specifically we fitted a model in which the CRP level for individual $i$ with RT variables $(RT_{ij})_{j=1}^{n}$ and lifestyle-medical and demographic (e.g., age, sex, race) variables $(Z_{ij})_{j=1}^{M}$was given by:

$\ln[CRP_{i}]=\sum_{j=1}^{M} \alpha_{j}Z_{ij}+\sum_{j=1}^{n} \beta_{j}RT_{ij}+\varepsilon_{i}$ (A2)

Preliminary analysis suggested that a logarithmic transformation of CRP concentrations satisfactorily normalized the residuals from the model, as determined by QQ plots and a Shapiro-Wilk normality test [^5^](#_ENREF_5), and this was therefore used in all subsequent analysis. The lifestyle-medical and demographic variables $(Z_{ij})$ comprised various explanatory variables other than RT, used to model CRP in the absence of radiation exposure; these variables are as discussed in the confounders section above and given in Supplementary Information Part A Table A1. In order to determine which of these background factors affected CRP in this cohort and to avoid the possibility of over-fitting, the Akaike Information Criterion (AIC) [^6^](#_ENREF_6),[^7^](#_ENREF_7) was used to select the optimal background model variables. A mixed forward-backward stepwise algorithm was used to select the variable set minimizing AIC, using R [^2^](#_ENREF_2). The final model was adjusted for the set of variables listed in Supplementary Information Part A Table A2. The missing indicator method was used to adjust for absent values of any covariates.

For preliminary descriptive analysis of the data, we examined differences in socio-demographic and clinical characteristics using three CRP risk categories recommended by the American Heart Association/Center for Disease Control as low risk (<1 mg/l), average risk (1-<3 mg/l), and high risk (≥3 mg/l); these are determined by levels of CRP in the adult population [^8^](#_ENREF_8). The percent change in CRP per RT cycle was computed as $100(e^{\beta_{j}}-1)$. [Note the non-linear nature of this function of $\beta_{j}$, so that model-predicted percentages for, say, two treatment cycles will not simply be this figure doubled.] All statistical analyses were carried out using ordinary least squares, and tests were performed using analysis of variance (ANOVA) [^9^](#_ENREF_9). For the descriptive analysis in Table 3 *p*-values were computed using Fisher’s exact test [^4^](#_ENREF_4), or, when this was not possible, using an approximation based on 10^7^ simulations. In the analyses of Table 4 and Figure 1, we give emphasis to analysing using the number of radiotherapy treatments (first questionnaire) or number of radiotherapy treatments or body regions exposed (second questionnaire); this uses the responses given by the technologists to the first two questionnaires, recording the regions treated, year first treated, and number of treatment cycles. For simplicity we term this measure the “numbers of RT treatments or body areas exposed to RT procedures”. The later questionnaires completed by the technologists did not elicit this information.

*Wording of questions asked in relation to disease endpoint and radiotherapy treatment*

*Questionnaire 1 (Q1) (1983-1989)*

1. Have you ever been told by a doctor that you had a thyroid condition, for example, thyroid nodules, thyroid cancer, hyperthyroidism, hypothyroidism, goiter, etc? What was the specific medical name for the thyroid condition(s)? [Mark all that apply] [The list of conditions includes:] (a) hyperthyroidism; (b) hypothyroidism. When was the [specified] thyroid condition first diagnosed (month, year)?

2. Have you ever personally undergone any therapeutic X-ray procedures? [(a) yes; (b) no; (c) don’t know] If you marked Yes, please mark the body site(s) treated with X-rays and the year treated, and list the reasons for the therapy. [The body sites listed were:] (a) head and neck; (b) pelvis; (c) extremities; (d) chest; (e) other body sites (with year first treated for each category)

*Questionnaire 2 (Q2) (1994-1998)*

1. Have you had any of the following conditions or procedures listed below diagnosed by a physician? [The list of conditions includes:] (a) arthritis, rheumatoid; (b) arthritis, other; (c) diabetes mellitus; (d) hyperthyroidism; (e) hypothyroidism (with year first diagnosed for each condition in the range: (a) <1980; (b) 1980-1984; (c) 1985-1989; (d) 1990+).

2. As a patient have you undergone any radiotherapy procedures, including radium implants or other brachytherapy? Please indicate whether you received radiotherapy to any of the following body areas for cancer or any other condition during the specified calendar years. If you did not receive radiotherapy during a specific time period leave items under that column blank. If you never received radiotherapy to a particular body area. mark the circle for 'Never received' and leave all other columns blank for that body area. [The list of body areas includes:] (a) head or neck; (b) shoulder; (c) chest or spine; (d) abdomen; (e) pelvis; (f) extremities; (g) other (specify). For each body site information is requested on period administered: (a) never received; (b) before 1980 (separately for cancer, not for cancer); (c) 1980-1989 (separately for cancer, not for cancer); (d) 1990+ (separately for cancer, not for cancer).

*Questionnaire 3 (Q3) (2003-2005)*

1. Did a doctor ever tell you that you had any of the following BENIGN tumors or other medical conditions? [The list of conditions includes:] (a) hyperthyroidism (overactive thyroid); (b) hypothyroidism (underactive thyroid) (and for each condition give year first diagnosed).

2. Did a doctor ever tell you that you had any of the following medical conditions? [The list of conditions includes:] (a) diabetes; (b) osteoarthritis; (c) rheumatoid arthritis (and for each condition give year first diagnosed).

**Supplementary Information Part B Table B1. Incidence of inflammatory disease following administration of radiotherapy in 110,368 U.S. radiologic technologists, with minimal adjustment for lifestyle and demographic factors.**^a^

|  | Hypothyroidism | | Hyperthyroidism | | Type-2 Diabetes | | Rheumatoid arthritis | | Osteoarthritis | |
| --- | --- | --- | --- | --- | --- | --- | --- | --- | --- | --- |
|  | 4725/74,165 |  | 1105/75,436 |  | 2557/50,135 |  | 759/50,227 |  | 4084/40,988 |  |
| Radiotherapy body regions received | RR (95% CI) | *p*-value^b^ | RR (95% CI) | *p*-value^b^ | RR (95% CI) | *p*-value^b^ | RR (95% CI) | *p*-value^b^ | RR (95% CI) | *p*-value^b^ |
| All radiotherapy (Q1) | 1.03 (0.94, 1.14) | 0.509 | 0.99 (0.81, 1.22) | 0.948 | 0.96 (0.87, 1.07) | 0.479 | 0.89 (0.75, 1.07) | 0.225 | 0.99 (0.91, 1.08) | 0.854 |
| All radiotherapy (Q1+Q2) | 1.15 (1.03, 1.28) | 0.019 | 1.12 (0.88, 1.41) | 0.366 | 1.05 (0.90, 1.21) | 0.535 | 0.84 (0.63, 1.13) | 0.239 | 1.21 (1.07, 1.36) | 0.003 |
| One vs None | 1.06 (0.91, 1.24) | 0.923 | 0.99 (0.71, 1.37) | 0.992 | 1.02 (0.84, 1.25) | 0.005 | 0.83 (0.56, 1.24) | 0.381 | 1.26 (1.07, 1.47) | 0.071 |
| Two vs None | 1.06 (0.72, 1.57) |  | 0.89 (0.37, 2.16) |  | 1.24 (0.80, 1.93)^c^ |  | 0.49 (0.12, 1.98) |  | 1.15 (0.75, 1.77) |  |
| Three vs None | 0.93 (0.35, 2.49) |  | 1.05 (0.15, 7.47) |  |  |  | 2.64 (0.66, 10.60) |  | 1.50 (0.62, 3.60) |  |
| Four vs None | 0.73 (0.18, 2.90) |  | 1.60 (0.23, 11.39) |  | 4.00 (1.66, 9.64) |  | 2.55 (0.36, 18.15) |  | 0.89 (0.22, 3.58) |  |
| Continuous trend per number of treatments | 1.03 (0.92, 1.14) | 0.648^d^ | 1.00 (0.80, 1.25) | 0.998^d^ | 1.11 (0.97, 1.26) | 0.126^d^ | 0.95 (0.72, 1.25) | 0.722^d^ | 1.15 (1.03, 1.28) | 0.015^d^ |

^a^Analysis is adjusted, for duration of work (year last worked – year first worked), and by stratification by sex and year of birth (<1900, 1900-1909, 1910-1919, 1920-1929, 1930-1939, 1940-1949, 1950-1959, 1960+). Unless otherwise indicated, all analysis uses only the responses to the first questionnaire (Q1) and not the second questionnaire (Q2).

^b^*p*-value of heterogeneity of relative risk, unless otherwise indicated;

^c^model with collapsed numbers of RT procedures: 0, 1, 2+3, 4;

^d^*p*-value of trend of relative risk with numbers of body regions receiving therapy.

**Supplementary Information Part B Table B2. Distribution of numbers of informative inflammatory disease incident cases / numbers in informative underlying population by whether or not the subjects reported history of radiotherapy among all technologists and subgroups according to demographic and lifestyle characteristics, 1983-89 or 1994-98 (baseline questionnaires) through 2008.**

| Characteristics | Hypothyroidism | | | Hyperthyroidism | | | Type-2 Diabetes | | | | | |
| --- | --- | --- | --- | --- | --- | --- | --- | --- | --- | --- | --- | --- |
|  | Yes | No |  | Yes | No |  | Yes | | No |  | | |
|  | RT / no RT | RT / no RT | *p*-value^a^ | RT / no RT | RT / no RT | *p*-value^a^ | RT / no RT | | RT / no RT | *p*-value^a^ | | |
| All technologists by inflammatory endpoint and receipt of radiotherapy (RT) | 338 / 4387 | 3995 / 65,445 | <0.001 | 78 / 1027 | 4392 / 69,939 | 0.109 | 199 / 2358 | | 2909 / 44,669 | 0.001 | | |
| Sex | | | | | | | | | | | |  |
| Male | 36 / 372 | 900 / 16,397 | <0.001 | 7 / 75 | 933 / 16,751 | <0.001 | 51 / 825 | | 413 / 8231 | <0.001 | | |
| Female | 302 / 4015 | 3095 / 49,048 |  | 71 / 952 | 3459 / 53,188 |  | 148 / 1533 | | 2496 / 36,438 |  | | |
| Race | | | | | | | | | | | |  |
| White | 330 / 4282 | 3854 / 62,090 | <0.001 | 71 / 982 | 4251 / 66,497 | 0.054 | 192 / 2158 | | 2851 / 42,941 | <0.001 | | |
| Black | 4 / 38 | 75 / 1798 |  | 3 / 26 | 75 / 1818 |  | 3 / 118 | | 30 / 913 |  | | |
| Asian + Pacific Islander | 2 / 23 | 30 / 748 |  | 2 / 10 | 31 / 763 |  | 2 / 39 | | 11 / 400 |  | | |
| Other/Unknown | 2 / 44 | 36 / 809 |  | 2 / 9 | 35 / 861 |  | 2 / 43 | | 17 / 415 |  | | |
| Birth year | | | | | | | | | | | |  |
| <1920 or missing | 5 / 37 | 194/801 | <0.001 | 1 / 7 | 209/853 | 0.036 | | 3 / 26 | 60/282 | | <0.001 |  |
| 1920-1929 | 32 / 159 | 506 / 2629 |  | 12 / 34 | 558 / 2832 |  | 24 / 136 | | 360 / 1628 |  | | |
| 1930-1939 | 75 / 510 | 920 / 7806 |  | 17 / 131 | 1013 / 8411 |  | 71 / 524 | | 689 / 5235 |  | | |
| 1940-1949 | 140 / 1552 | 1340 / 21,382 |  | 26 / 323 | 1501 / 23,074 |  | 75 / 942 | | 1032 / 14,942 |  | | |
| 1950-1959 | 85 / 2088 | 1023 / 32,240 |  | 20 / 519 | 1100 / 34,152 |  | 26 / 725 | | 761 / 22,164 |  | | |
| 1960+ | 1 / 41 | 12 / 587 |  | 2 / 13 | 11 / 617 |  | 0 / 5 | | 7 / 418 |  | | |
| Attained age at diagnosis | | | | | | | | | | | |  |
| <50 | 3 / 137 | 58 / 2441 | <0.001 | 2 / 38 | 60 / 2562 | 0.386 | 1 / 29 | | 30 / 1573 | <0.001 | | |
| 50-59 | 101 / 2221 | 1179 / 33,966 |  | 24 / 539 | 1276 / 36,023 |  | 33 / 822 | | 861 / 23,232 |  | | |
| 60-69 | 127 / 1418 | 1356 / 19,433 |  | 27 / 297 | 1499 / 21,012 |  | 77 / 900 | | 1012 / 13,534 |  | | |
| 70-79 | 80 / 462 | 874 / 7075 |  | 17 / 123 | 974 / 7618 |  | 67 / 479 | | 656 / 4764 |  | | |
| 80+ | 27 / 149 | 528 / 2520 |  | 8 / 30 | 583 / 2724 |  | 21 / 128 | | 350 / 1566 |  | | |
| Smoking status | | | | | | | | | | | |  |
| Non-Smoker | 148 / 2225 | 1698 / 32,120 | 0.003 | 30 / 458 | 1878 / 34,428 | <0.001 | 86 / 1045 | | 1276 / 22,823 | <0.001 | | |
| Former Smoker | 112 / 1175 | 1287 / 18,296 |  | 28 / 263 | 1419 / 19,593 |  | 62 / 699 | | 976 / 12,390 |  | | |
| Current Smoker | 75 / 948 | 951 / 14,503 |  | 19 / 300 | 1036 / 15,352 |  | 51 / 587 | | 621 / 9169 |  | | |
| Unknown Smoking status | 3 / 39 | 59 / 526 |  | 1 / 6 | 59 / 566 |  | 0 / 27 | | 36 / 287 |  | | |
| Body mass index (kg m^-2^) | | | | | | | | | | | |  |
| Missing | 9 / 126 | 122 / 1570 | <0.001 | 2 / 19 | 130 / 1705 | <0.001 | 8 / 92 | | 75 / 975 | <0.001 | | |
| <18.5 | 9 / 128 | 107 / 2079 |  | 1 / 54 | 117 / 2175 |  | 1 / 16 | | 87 / 1429 |  | | |
| 18.5-24.9 | 224 / 2946 | 2413 / 41,849 |  | 52 / 703 | 2652 / 44,597 |  | 52 / 627 | | 1881 / 29,919 |  | | |
| 25.0-29.9 | 66 / 853 | 977 / 14,906 |  | 12 / 178 | 1078 / 15,916 |  | 85 / 952 | | 642 / 9439 |  | | |
| ≥30.0 | 30 / 334 | 376 / 5041 |  | 11 / 73 | 415 / 5546 |  | 53 / 671 | | 224 / 2907 |  | | |

^a^*p*-value of heterogeneity, evaluated via Fisher’s exact test, or a Monte Carlo analogue.

| Characteristics | Rheumatoid arthritis | | | Osteoarthritis | | | | |
| --- | --- | --- | --- | --- | --- | --- | --- | --- |
|  | Yes | No |  | Yes | No | |  | |
|  | RT / no RT | RT / no RT | *p*-value^a^ | RT / no RT | RT / no RT | | *p*-value^a^ | |
| All technologists by inflammatory endpoint and receipt of radiotherapy (RT) | 50 / 709 | 3038 / 46,430 | 0.594 | 292 / 3792 | 1722 / 35182 | <0.001 | |  |
| Sex | | | | | | | |  |
| Male | 4 / 105 | 460 / 9292 | 0.002 | 19 / 392 | 298 / 7534 | <0.001 | |  |
| Female | 46 / 604 | 2578 / 37,138 |  | 273 / 3400 | 1424 / 27,648 |  | |  |
| Race |  |  |  |  |  |  | |  |
| White | 48 / 679 | 2968 / 44,472 | 0.366 | 285 / 3673 | 1682 / 33,563 | <0.001 | |  |
| Black | 2 / 19 | 36 / 1030 |  | 2 / 85 | 19 / 806 |  | |  |
| Asian + Pacific Islander | 0 / 3 | 16 / 457 |  | 2 / 16 | 8 / 399 |  | |  |
| Other/Unknown | 0 / 8 | 18 / 471 |  | 3 / 18 | 13 / 414 |  | |  |
| Birth year | | | | | | | |  |
| <1920 | 1 / 4 | 56 / 290 | <0.001 | 0 / 5 | 2 / 20 | <0.001 | |  |
| 1920-1929 | 9 / 50 | 368 / 1724 |  | 6 / 47 | 39 / 357 |  | |  |
| 1930-1939 | 15 / 124 | 747 / 5722 |  | 76 / 534 | 371 / 3452 |  | |  |
| 1940-1949 | 15 / 286 | 1084 / 15,649 |  | 153 / 1671 | 677 / 11,354 |  | |  |
| 1950-1959 | 10 / 242 | 776 / 22,626 |  | 57 / 1521 | 626 / 19,608 |  | |  |
| 1960+ | 0 / 3 | 7 / 419 |  | 0 / 14 | 7 / 391 |  | |  |
| Attained age at diagnosis | | | | | | | |  |
| <=50 | 0 / 12 | 30 / 1590 | <0.001 | 3 / 58 | 24 / 1483 | <0.001 | |  |
| 50-59 | 10 / 266 | 886 / 23,778 |  | 67 / 1718 | 702 / 20,355 |  | |  |
| 60-69 | 17 / 269 | 1064 / 14,235 |  | 156 / 1522 | 645 / 10,085 |  | |  |
| 70-79 | 14 / 114 | 708 / 5203 |  | 63 / 459 | 318 / 2981 |  | |  |
| 80+ | 9 / 48 | 350 / 1624 |  | 3 / 35 | 33 / 278 |  | |  |
| Smoking status | | | | | | | |  |
| Non-Smoker | 22 / 293 | 1345 / 23,593 | <0.001 | 124 / 1860 | 779 / 18,408 | <0.001 | |  |
| Former Smoker | 16 / 209 | 1009 / 12,989 |  | 101 / 1111 | 535 / 9313 |  | |  |
| Current Smoker | 11 / 200 | 652 / 9538 |  | 63 / 803 | 393 / 7244 |  | |  |
| Unknown Smoking status | 1 / 7 | 32 / 310 |  | 4 / 18 | 15 / 217 |  | |  |
| Body mass index (kg m^-2^) | | | | | | | |  |
| Missing | 1 / 22 | 84 / 1050 | <0.001 | 9 / 85 | 44 / 758 | <0.001 | |  |
| <18.5 | 0 / 21 | 85 / 1404 |  | 5 / 90 | 59 / 1182 |  | |  |
| 18.5-24.9 | 27 / 382 | 1853 / 29,789 |  | 189 / 2347 | 1132 / 23,539 |  | |  |
| 25.0-29.9 | 18 / 201 | 715 / 10,358 |  | 60 / 832 | 359 / 7386 |  | |  |
| ≥30.0 | 4 / 83 | 301 / 3829 |  | 29 / 438 | 128 / 2317 |  | |  |

^a^*p*-value of heterogeneity, evaluated via Fisher’s exact test, or a Monte Carlo analogue.

**Supplementary Information Part B Table B3. Incidence of cases of inflammatory disease (hypothyroidism, hyperthyroidism, diabetes, rheumatoid arthritis, osteoarthritis) in relation to body part of administration of radiotherapy, or reason for treatment with radiotherapy (as a patient) among 110,368 US Radiologic Technologists.^a^**

|  | Hypothyroidism (*n*=4725) | | Hyperthyroidism (*n*=1105) | | Type-2 Diabetes (*n*=2557) | | Rheumatoid arthritis (*n*=759) | | Osteoarthritis (*n*=4084) | |
| --- | --- | --- | --- | --- | --- | --- | --- | --- | --- | --- |
|  | Relative risk (+95% CI) | *p*-value | Relative risk (+95% CI) | *p*-value | Relative risk (+95% CI) | *p*-value | Relative risk (+95% CI) | *p*-value | Relative risk (+95% CI) | *p*-value |
| Radiotherapy to head (Q1 only) | 1.07 (0.85, 1.34) | 0.589 | 1.18 (0.75, 1.84) | 0.487 | 1.20 (0.91, 1.59) | 0.208 | 0.46 (0.22, 0.97) | 0.019 | 1.34 (1.08, 1.68) | 0.012 |
| Radiotherapy to head | 1.18 (0.97, 1.43) | 0.102 | 1.18 (0.79, 1.75) | 0.440 | 0.98 (0.76, 1.26) | 0.862 | 0.61 (0.35, 1.06) | 0.060 | 1.28 (1.05, 1.55) | 0.017 |
| Radiotherapy to pelvis (Q1 only) | 0.64 (0.33, 1.24) | 0.154 | 1.15 (0.43, 3.10) | 0.786 | 1.23 (0.64, 2.39) | 0.550 | 2.08 (0.85, 5.07) | 0.148 | 1.11 (0.61, 2.01) | 0.740 |
| Radiotherapy to pelvis | 0.78 (0.46, 1.31) | 0.325 | 1.57 (0.74, 3.34) | 0.270 | 1.20 (0.69, 2.09) | 0.531 | 2.20 (1.04, 4.67) | 0.068 | 1.11 (0.67, 1.84) | 0.697 |
| Radiotherapy to chest (Q1 only) | 0.97 (0.69, 1.37) | 0.870 | 0.92 (0.46, 1.85) | 0.811 | 1.21 (0.80, 1.84) | 0.378 | 1.34 (0.63, 2.82) | 0.469 | 1.28 (0.90, 1.82) | 0.185 |
| Radiotherapy to chest | 1.05 (0.78, 1.42) | 0.741 | 1.06 (0.58, 1.92) | 0.859 | 1.36 (0.96, 1.94) | 0.102 | 1.14 (0.57, 2.30) | 0.719 | 1.21 (0.87, 1.68) | 0.261 |
| Radiotherapy to extremity (Q1 only) | 1.05 (0.88, 1.26) | 0.568 | 0.84 (0.56, 1.28) | 0.404 | 1.16 (0.91, 1.47) | 0.236 | 1.07 (0.68, 1.67) | 0.768 | 1.11 (0.91, 1.36) | 0.308 |
| Radiotherapy to extremity | 1.02 (0.86, 1.21) | 0.863 | 0.92 (0.63, 1.33) | 0.644 | 1.09 (0.87, 1.36) | 0.462 | 0.94 (0.61, 1.44) | 0.772 | 1.12 (0.93, 1.34) | 0.229 |
| Radiotherapy to shoulder (Q2 only) | 0.74 (0.41, 1.34) | 0.296 | 1.30 (0.53, 3.20) | 0.577 | 1.01 (0.55, 1.85) | 0.969 | 0.93 (0.29, 2.92) | 0.896 | 1.08 (0.52, 2.24) | 0.835 |
| Radiotherapy to abdomen (Q2 only) | 1.32 (0.69, 2.55) | 0.425 | 2.96 (1.21, 7.20) | 0.042 | 1.71 (0.85, 3.47) | 0.168 | 2.27 (0.72, 7.11) | 0.215 | 1.26 (0.59, 2.66) | 0.567 |
| Radiotherapy to other part (Q2 only)^b^ | 0.94 (0.64, 1.36) | 0.730 | 0.96 (0.45, 2.03) | 0.913 | 1.19 (0.79, 1.80) | 0.424 | 1.41 (0.72, 2.73) | 0.339 | 0.99 (0.67, 1.46) | 0.950 |
| Radiotherapy for cancer (Q2 only) | 1.10 (0.68, 1.78) | 0.689 | 1.89 (0.89, 4.00) | 0.133 | 1.59 (1.01, 2.52) | 0.063 | 1.64 (0.73, 3.70) | 0.265 | 1.27 (0.79, 2.02) | 0.341 |
| Radiotherapy for non-cancer (Q2 only) | 1.09 (0.92, 1.30) | 0.321 | 1.27 (0.91, 1.78) | 0.170 | 0.93 (0.75, 1.16) | 0.533 | 0.77 (0.50, 1.19) | 0.220 | 1.25 (1.06, 1.48) | 0.013 |

^a^The relative risk and *p*-values are obtained via fitting a Cox model with age as timescale and adjusted, for duration of work (year last worked – year first worked), and via stratification by sex, year of birth (<1900, 1900-1909, 1910-1919, 1920-1929, 1930-1939, 1940-1949, 1950-1959, 1960+), body mass index (<18.5 or missing, 18.5-24.9, 25.0-29.9, 30.0+ kg m^-2^), smoking status (never, former, current smoker) and racial group (white, black, Asian, other/unknown). Unless otherwise indicated, all analysis uses the responses [in relation to body part treated, or reason for treatment] to the first questionnaire (Q1) and the second questionnaire (Q2).

^b^other than to head/neck, shoulder, chest/spine, abdomen, pelvis, extremities.

**Supplementary Information Part B Table B4. Radiotherapy and subsequent CRP according to demographic and lifestyle factors, among 1326 US Radiologic Technologists with a usable blood draw sample.^a^**

| Characteristics | Missing or CRP <1 mg/l | |  | | CRP 1-<3 mg/l | |  | CRP ≥ 3mg/l | *p*-value^b^ |
| --- | --- | --- | --- | --- | --- | --- | --- | --- | --- |
|  | Radiotherapy / no radiotherapy | |  | | Radiotherapy / no radiotherapy | |  | Radiotherapy / no radiotherapy |  |
| Total technologists | 11 / 292 | |  | | 24 / 419 | |  | 31 / 549 | 0.481 |
| Sex | | | | | | | | | |
| Female | | 7 / 168 | |  | | 12 / 248 | |  | 23 / 365 | 0.035 |
| Male | 4 / 124 | |  | | 12 / 171 | |  | 8 / 184 |  |
| Race | | | | | | | | | | |
| White | | | 10 / 196 | |  | | 21 / 261 |  | 19 / 299 | <0.001 |
| Black | | | 0 / 93 | |  | | 3 / 155 |  | 12 / 248 |  |
| Asian + Pacific Islanders | | | 1 / 1 | |  | | 0 / 0 |  | 0 / 1 |  |
| Other/Unknown | | | 0 / 2 | |  | | 0 / 3 |  | 0 / 1 |  |
| Birth year | | | | | | | | | | |
| <1920 | | | 1 / 3 | |  | | 2 / 6 |  | 2 / 9 | 0.887 |
| 1920-1929 | | | 5 / 32 | |  | | 7 / 47 |  | 6 / 53 |  |
| 1930-1939 | | | 3 / 64 | |  | | 6 / 97 |  | 11 / 134 |  |
| 1940-1949 | | | 1 / 67 | |  | | 5 / 116 |  | 9 / 147 |  |
| 1950+ | | | 1 / 126 | |  | | 4 / 153 |  | 3 / 206 |  |
| Attained age when CRP measured | | | | | | | | | | |
| <50 | | | 0 / 7 | |  | | 0 / 3 |  | 0 / 6 | 0.906 |
| 50-59 | | | 1 / 124 | |  | | 4 / 169 |  | 4 / 220 |  |
| 60-69 | | | 1 / 67 | |  | | 7 / 105 |  | 10 / 132 |  |
| 70-79 | | | 7 / 74 | |  | | 9 / 115 |  | 12 / 153 |  |
| 80+ | | | 2 / 20 | |  | | 4 / 27 |  | 5 / 38 |  |
| Smoking status | | | | | | | | | | |
| Missing | | | 6 / 23 | |  | | 7 / 42 |  | 9 / 57 | 0.233 |
| Not current smoker | | | 5 / 266 | |  | | 17 / 365 |  | 19 / 473 |  |
| Current smoker | | | 0 / 3 | |  | | 0 / 12 |  | 3 / 19 |  |
| Body mass index (kg m^-2^) | | | | | | | | | | |
| Unknown | | | 0 / 59 | |  | | 1 / 83 |  | 2 / 105 | <0.001 |
| < 18.5 | | | 0 / 7 | |  | | 1 / 9 |  | 0 / 4 |  |
| 18.5-24.9 | | | 5 / 162 | |  | | 11 / 196 |  | 15 / 228 |  |
| 25.0-29.9 | | | 5 / 46 | |  | | 9 / 103 |  | 7 / 150 |  |
| 30+ | | | 1 / 18 | |  | | 2 / 28 |  | 7 / 62 |  |

^a^Radiotherapy (presence or absence) is determined using all information from the first and second questionnaires.

^b^*p*-value of heterogeneity, evaluated via Fisher’s exact test, or a Monte Carlo analogue

**References**

1 Cox, D. R. Regression models and life-tables. *J. Royal Statist. Soc. Series B* **34**, 187-220 (1972).

2 R: A language and environment for statistical computing. version 3.4.4 <https://www.r-project.org> v. 3.4.4 (R Foundation for Statistical Computing, Vienna, Austria, 2018).

3 McCullagh, P. & Nelder, J. A. in *Monographs on statistics and applied probability 37* 1-526 (Chapman and Hall/CRC, Boca Raton, FL, 1989).

4 Fisher, R. A. On the interpretation of χ^2^ from contingency tables, and the calculation of P. *J. Roy. Statist. Soc.* **85**, 87-94 (1922).

5 Shapiro, S. S. & Wilk, M. B. in *Biometrika* Vol. 52 591-611 (1965).

6 Akaike, H. in *2nd International Symposium on Information Theory* (eds B.N. Petrov & F. Czáki) 267-281 (Akadémiai Kiadó, Budapest, 1973).

7 Akaike, H. Likelihood of a model and information criteria. *J. Econometrics* **16**, 3-14 (1981).

8 Pearson, T. A. *et al.* Markers of inflammation and cardiovascular disease: application to clinical and public health practice: A statement for healthcare professionals from the Centers for Disease Control and Prevention and the American Heart Association. *Circulation* **107**, 499-511 (2003).

9 Rao, C. R. *Linear statistical inference and its applications. 2nd edition*. (John Wiley & Sons, Inc, 2002).
